# Modulation of the microbiota-lipid-brain axis by modified Chaihu-Longgu-Muli Decoction ameliorates chronic stress-induced depression

**DOI:** 10.3389/fnmol.2026.1865663

**Published:** 2026-07-02

**Authors:** Yonghao Chen, Yongcheng Liu, Ruxi Tong, Lizhen Lin, Ming Zhu, Xuanbin Huang, Tianmin Wu

**Affiliations:** 1 Department of Traditional Chinese Medicine, The First Affiliated Hospital, Fujian Medical University, Fuzhou, China; 2 National Regional Medical Center, Binhai Campus of the First Affiliated Hospital, Fujian Medical University, Fuzhou, China

**Keywords:** chronic unpredictable mild stress, major depressive disorder, microbiota-lipid-brain axis, modified Chaihu-Longgu-Muli Decoction, multi-omics

## Abstract

**Background and aims:**

Modified Chaihu-Longgu-Muli Decoction (mCLMD) has well-established clinical antidepressant efficacy, yet its precise systemic mechanisms of action remain incompletely characterized. Here, we employed an integrated multi-omics strategy to delineate the mechanisms by which mCLMD exerts antidepressant-like effects via modulation of the gut microbiota-lipid-brain axis.

**Experimental procedures:**

Rats exposed to chronic unpredictable mild stress (CUMS) were administered graded doses of mCLMD. Depressive-like behaviors were assessed using the sucrose preference test, forced swim test, and open field test. To delineate the underlying mechanisms, we integrated network pharmacology analysis, 16S rRNA gene sequencing, serum metabolomics, and targeted validation of hippocampal signaling pathways.

**Results:**

mCLMD administration dose-dependently reversed CUMS-induced depressive-like behaviors in rats. Consistent with network pharmacology predictions, 16S rRNA sequencing revealed that mCLMD ameliorated CUMS-induced gut dysbiosis, characterized by reduced relative abundance of pro-inflammatory genera (*Colidextribacter*, *Oscillibacter*) and enrichment of beneficial taxa (*Romboutsia*, *Lactobacillus*). Metabolomic profiling demonstrated concomitant restoration of dysregulated lipid and neurosteroid profiles in serum. Correlation analysis identified that reduced abundance of stress-associated pathobionts was tightly linked to decreased levels of peripherally derived neurosteroids with neurotoxic potential (e.g., pregnenolone), while enrichment of beneficial commensals correlated with elevated levels of neuroprotective endocannabinoid precursors (including 1-stearoyl-2-arachidonoylglycerol). These peripheral immunometabolic alterations were accompanied by the transcriptional upregulation of the hippocampal cAMP-BDNF–TrkB signaling pathway and restoration of monoaminergic neurotransmission.

**Conclusion:**

The antidepressant effects of mCLMD are strongly associated with systemic remodeling of the gut microbiota-lipid-brain axis. These therapeutic effects potentially stem from both the direct pharmacological activity of mCLMD’s bioactive compounds and indirect modulation of the gut microbiota and host metabolism. Collectively, our findings provide robust preclinical evidence underpinning the clinical application of mCLMD in the management of major depressive disorder.

## 1 Introduction

Major depressive disorder (MDD) is a chronic, relapsing psychiatric disorder affecting over 280 million people worldwide and the leading cause of years lived with disability globally (Rong et al., 2025). For more than half a century, first-line antidepressant therapies have been based on the monoamine deficiency hypothesis. However, this classical framework cannot fully explain the 2–4 week delay in therapeutic onset or the ~30% rate of treatment resistance seen in clinical practice (Cipriani et al., 2018; Ding et al., 2024). Mounting evidence now demonstrates that MDD pathogenesis is not limited to the central nervous system but involves systemic disturbances including neuroinflammation, hypothalamic–pituitary–adrenal (HPA) axis hyperactivity, and widespread metabolic dysregulation (Ding et al., 2024). These interconnected pathologies highlight the need for safer, multi-target therapeutic strategies.

Traditional Chinese medicine (TCM), with its holistic philosophy and multi-component regulatory effects, represents a valuable resource for developing novel antidepressants. Chaihu-Longgu-Muli Decoction (CLMD) is a classical herbal formula that has been used clinically for centuries to manage anxiety, insomnia, and depressive symptoms. Numerous preclinical studies and meta-analyses consistently show that CLMD exerts significant neuroprotective effects—including reducing neuroinflammation and upregulating hippocampal brain-derived neurotrophic factor (BDNF)—and enhances efficacy while reducing side effects when combined with selective serotonin reuptake inhibitors (Wang et al., 2019; Jia et al., 2023).

Building on this work, we developed a modified formulation of CLMD (mCLMD) by integrating modern pharmacological knowledge with clinical experience. This optimized formula incorporates botanicals with well-documented neuroprotective, anxiolytic, and prebiotic properties—including *Bulbus Lilii*, *Radix Rehmanniae*, *Radix Polygalae*, and *Sclerotium Poriae Pararadicis*—while omitting components with redundant or non-essential effects. These modifications were designed to synergistically enhance BDNF/TrkB signaling (Wang et al., 2024; Wang et al., 2025), modulate monoaminergic and GABAergic neurotransmission (Chen et al., 2023; Zhou et al., 2025), and maintain gut microbial homeostasis (Du et al., 2020; Liu et al., 2024; Yan et al., 2025).

Despite its promising clinical efficacy, the systemic mechanisms underlying mCLMD’s antidepressant effects remain poorly understood. While network pharmacology studies have provided a preliminary framework pointing to a “periphery-to-central” mode of action, rigorous *in vivo* validation using multi-omics approaches is needed to identify the specific biological pathways linking peripheral immunometabolic changes to central neuroplasticity (Zhao et al., 2024).

The microbiota-gut-brain axis has emerged as a key paradigm in understanding MDD pathogenesis. Gut dysbiosis triggers both systemic immune activation and metabolic dysregulation (Yang et al., 2020; Hao et al., 2024) Importantly, the gut microbiome is now recognized as a major regulator of systemic lipid mediators and neurosteroid biosynthesis. Microbiota-driven alterations in circulating endocannabinoid precursors and neurosteroids can directly influence CNS function by crossing the blood–brain barrier and modulating hippocampal neuroplasticity via the cAMP-BDNF–TrkB pathway, which is significantly suppressed during chronic stress (Casarotto et al., 2021; Markov and Novosadova, 2022).

Based on these findings, we hypothesized that mCLMD exerts antidepressant-like effects by restructuring the gut microbiota and correcting systemic metabolic abnormalities, particularly in lipid and neurosteroid pathways. To test this hypothesis, we used an integrated approach combining network pharmacology, 16S rRNA gene sequencing, UPLC-MS/MS untargeted metabolomics, and targeted molecular validation of hippocampal signaling pathways in a rat model of chronic unpredictable mild stress (CUMS). Our findings offer insights into the systemic mechanisms of mCLMD, supporting its clinical application in the treatment of major depressive disorder.

## 2 Materials and methods

### 2.1 Preparation of modified Chaihu-Longgu-Muli Decoction (mCLMD)

Modified Chaihu-Longgu-Muli Decoction (mCLMD) is derived from the classical formula recorded in the *Treatise on Cold Damage Disorders*. To optimize its antidepressant efficacy, we made three key modifications: addition of six herbs (*Bulbus Lilii*, *Radix Rehmanniae*, *Radix Polygalae*, *Semen Ziziphi Spinosae*, *Fructus Tritici Levis*, and *Rhizoma Chuanxiong*), replacement of *Poria* with *Sclerotium Poriae Pararadicis*, and substitution of *Ginseng Radix* with *Radix Codonopsis*. This formulation follows the core TCM therapeutic principle of “soothing the liver, relieving depression, nourishing the heart and calming the mind”, which addresses the core TCM pathogenesis of depression: liver qi stagnation with heart-spleen deficiency. The optimized formula was specifically designed to regulate neuroinflammation, promote hippocampal neuroplasticity, and exert antidepressant effects. It comprises 15 herbal and mineral ingredients, with full details of composition and dosage listed in Table 1.

**Table 1:** Composition and dosage of mCLMD.

Chinese name	Latin botanical name	Medicinal name (Pharmacopoeia)	Weight (g)
Chai Hu	*Bupleurum chinense* DC.	*Radix Bupleuri*	10
Gui Zhi	*Cinnamomum cassia* Presl	*Ramulus Cinnamomi*	10
Long Gu	*Fossilia Ossis Mastodi*	Os Draconis (Fossilized bones)	20
Mu Li	*Ostrea gigas* Thunberg	Concha Ostreae	20
Bai He	*Lilium lancifolium* Thunb.	*Bulbus Lilii*	20
Sheng Di Huang	*Rehmannia glutinosa* Libosch.	*Radix Rehmanniae*	15
Huang Qin	*Scutellaria baicalensis* Georgi	*Radix Scutellariae*	10
Ban Xia	*Pinellia ternata* (Thunb.) Breit.	*Rhizoma Pinelliae Praeparatum*	10
Suan Zao Ren	*Ziziphus jujuba* Mill. var. *spinosa*	*Semen Ziziphi Spinosae*	15
Yuan Zhi	*Polygala tenuifolia* Willd.	*Radix Polygalae*	8
Fu Xiao Mai	*Triticum aestivum* L.	*Fructus Tritici Levis*	30
Gan Cao	*Glycyrrhiza uralensis* Fisch.	*Radix Glycyrrhizae*	5
Dang Shen	*Codonopsis pilosula* (Franch.) Nannf.	*Radix Codonopsis*	15
Fu Shen	*Poria cocos* (Schw.) Wolf	*Sclerotium Poriae Pararadicis*	15
Chuan Xiong	*Ligusticum chuanxiong* Hort.	*Rhizoma Chuanxiong*	10

All crude medicinal materials were authenticated by a licensed TCM pharmacist and obtained from the Department of Traditional Chinese Medicine Pharmacy, First Affiliated Hospital of Fujian Medical University. Mineral ingredients (Os Draconis and Concha Ostreae) were crushed and decocted alone for 30 min prior to adding the remaining herbs. The decoction was prepared according to the water extraction guidelines for TCM formulas in the *Pharmacopoeia of the People’s Republic of China* (2020 Edition), with minor adjustments based on classical literature for the original Chaihu-Longgu-Muli Decoction. Briefly, the combined herbs were soaked in 10 volumes of distilled water (v/w) for 30 min and extracted twice under reflux for 1 h each time. The combined filtrates were concentrated under reduced pressure at 60 °C using a rotary evaporator to a final concentration of 2.22 g crude drug/mL.

#### 2.1.1 Quality control

All raw materials complied with the quality and safety standards of the *Pharmacopoeia of the People’s Republic of China* (2020 Edition). The extraction process was strictly standardized across all batches to ensure consistent extraction efficiency. All batches were tested for heavy metals and arsenic, with results within acceptable safety limits. Stock extracts were stored at 4 °C and diluted to the desired concentrations with distilled water immediately prior to intragastric administration.

### 2.2 Network pharmacology analysis

#### 2.2.1 Active ingredients and target acquisition

We retrieved active compounds for each herb in mCLMD from the TCMSP and HERB databases (data retrieved January 2026). For ingredients not covered in these databases, we supplemented 14 additional antidepressant-active components identified through a systematic literature review. Compounds were screened using standard thresholds of oral bioavailability (OB) ≥ 30% and drug-likeness (DL) ≥ 0.18, the standard criteria for bioactive component identification in TCM network pharmacology studies (Ru et al., 2014). All compounds with confirmed toxicity were excluded from further analysis. We collected corresponding targets for the screened active compounds and converted them to official human gene symbols using the UniProtKB database (release 2026_01). We used human targets because core pathogenic pathways in depression and their gene orthologs are highly evolutionarily conserved between *Homo sapiens* and *Rattus norvegicus* (average sequence identity > 85% for the neurotrophic signaling and monoaminergic neurotransmission pathways investigated in this study).

#### 2.2.2 Depression-related target retrieval

We retrieved depression-associated targets from five public databases: GeneCards (relevance score ≥ 3), OMIM, TTD, DisGeNet, and DrugBank, using the keywords “major depressive disorder” and “depression.” Duplicates were removed after merging all datasets.

#### 2.2.3 Network construction and core target screening

We identified overlapping targets between mCLMD active compounds and depression-related targets using a Venn diagram. We constructed a protein–protein interaction (PPI) network of the overlapping targets using the STRING database (confidence score > 0.90, species: *Homo sapiens*, disconnected nodes removed). We selected this high-confidence threshold to balance network comprehensiveness and biological reliability. The PPI network was visualized in Cytoscape (v3.10). We screened core hub targets using the CytoNCA plugin via two rounds of median filtering based on four centrality metrics: degree, betweenness, closeness, and eigenvector centrality.

#### 2.2.4 GO and KEGG enrichment analysis

We performed Gene Ontology (GO) functional annotation and Kyoto Encyclopedia of Genes and Genomes (KEGG) pathway enrichment analysis on the core hub targets using the clusterProfiler package in R (v4.5.3). An adjusted *p*-value < 0.05 (Benjamini-Hochberg correction) was set as the statistical significance threshold.

### 2.3 Animals, ethical statement, and welfare monitoring

Male Sprague–Dawley rats (6–8 weeks, 200 ± 20 g) were housed under standard SPF conditions. Procedures were approved by the IACUC of Jiangxi Zhonghong Boyuan Biological Technology Co., Ltd. (K-2024-0740-1) and adhered to NIH guidelines and the 3Rs principles. *In vivo* models were irreplaceable for studying systemic microbiota-brain interactions (Replacement). Sample sizes (n = 6/group) were statistically minimized for exploratory multi-omics screening (Reduction). Trained staff conducted daily welfare monitoring of body weight and behavior (Refinement). Humane endpoints (>20% weight loss, self-mutilation, or resource inaccessibility) were established; no animals reached these endpoints or died prematurely. Rats failing initial modeling criteria (sucrose preference <60%) were excluded pre-treatment. As terminal multi-organ collection precluded rehoming, rats were deeply anesthetized (2% sodium pentobarbital, 40 mg/kg, i.p.) and humanely euthanized via cervical dislocation per AVMA guidelines.

### 2.4 Experimental design and drug administration

After a 1-week acclimation period, we randomly assigned rats to 6 groups (n = 6 per group) using a random number table:

1. Control group (CON): normal housing + intragastric administration of distilled water.
2. Model group (MOD): CUMS exposure + distilled water
3. Positive control group (VEN): CUMS exposure + venlafaxine hydrochloride at 15.63 mg/kg/day.
4. Low-dose mCLMD group (CL): CUMS exposure + mCLMD at 11.09 g/kg/day.
5. Medium-dose mCLMD group (CM): CUMS exposure + mCLMD at 22.19 g/kg/day.
6. High-dose mCLMD group (CH): CUMS exposure + mCLMD at 44.38 g/kg/day.

The medium mCLMD dose was converted from the standard clinical adult dose (213 g crude drug per 60 kg body weight) using body surface area normalization; low and high doses were set at 0.5 × and 2 × the medium dose, respectively.

All rats first underwent 4 weeks of CUMS exposure to establish the depressive-like model. We confirmed successful model establishment using the sucrose preference test (SPT) at the end of the 4-week CUMS period; only rats with sucrose preference < 60% relative to the CON group mean were included in the subsequent 4-week treatment phase.

All treatments were administered once daily via intragastric gavage at a constant volume of 10 mL/kg body weight for 4 consecutive weeks, between 9:00 and 10:00 a.m. The CUMS procedure continued for a total of 8 weeks: 4 weeks for model establishment, followed by 4 weeks of concurrent treatment and stress exposure (Markov and Novosadova, 2022). We applied 7 unpredictable mild stressors in random order (1–2 stressors per day, with no identical stressor repeated within 72 h to maintain unpredictability): 24-h food deprivation, 24-h water deprivation, 12-h reversed light/dark cycle, 24-h wet cage bedding, 45° cage tilt for 12 h, 30-min white noise exposure (85 dB), and 1-min tail clamping (1 cm from the tail tip). All stressors were applied between 8:00 a.m. and 6:00 p.m.

After the 4-week treatment period, we performed behavioral tests in the following order: SPT, open-field test (OFT), and forced swim test (FST). A 24-h recovery interval was allowed between tests to minimize carry-over effects. To balance mechanistic depth with sequencing and metabolomics costs, we prioritized the high-dose group because it exhibited the most robust and consistent behavioral efficacy across all behavioral paradigms. The present study was therefore designed primarily to identify core therapeutic mechanisms associated with maximal pharmacodynamic response rather than to establish dose–response omics relationships.

At the end of the experiment, we deeply anesthetized rats with 2% sodium pentobarbital (40 mg/kg, intraperitoneal injection). We collected blood from the abdominal aorta within 5 min of anesthesia induction, separated serum by centrifugation at 3000 × g for 15 min at 4 °C, and stored samples at −80 °C for untargeted metabolomics analysis. Rats were then euthanized by cervical dislocation in accordance with AVMA guidelines. We rapidly isolated whole brains on ice within 3 min of euthanasia and dissected bilateral hippocampi for molecular assays. We aseptically collected colonic fecal contents from the distal colon for 16S rRNA gene sequencing. All samples were snap-frozen in liquid nitrogen immediately after collection and stored at −80 °C until analysis.

Blinding and randomization: Allocation concealment was achieved using sequentially numbered, opaque sealed envelopes. Behavioral testing, sample processing, and all omics data analyses were performed by investigators blinded to group assignments. Furthermore, the order of sample processing and instrument injection for all molecular assays and omics analyses was fully randomized to minimize potential batch effects.

### 2.5 Behavioral tests

All behavioral tests were performed in a sound-attenuated, temperature-controlled room (22 ± 2 °C). All experimenters were blinded to group assignments throughout testing, and all apparatuses were thoroughly cleaned with 75% ethanol between animals to eliminate residual olfactory cues.

#### 2.5.1 Sucrose preference test (SPT)

The SPT was used to assess anhedonia, a core depressive-like phenotype. Rats were first habituated to two pre-weighed water bottles for 48 h, with bottle positions exchanged every 24 h to prevent position bias. Following 16 h of food and water deprivation, each rat was given simultaneous free access to two pre-weighed bottles: one containing 1% (w/v) sucrose solution and the other containing distilled water, for a 1-h test period. Sucrose preference was calculated as: (sucrose intake / (sucrose intake + water intake)) × 100%.

#### 2.5.2 Open-field test (OFT)

The OFT was used to assess locomotor activity and anxiety-like behavior. We gently placed each rat in the center of a black square arena (100 × 100 × 40 cm) and allowed it to explore freely for 5 min. We recorded the total distance traveled and the time spent in the central zone (50 × 50 cm, defined as the area more than 25 cm away from any wall) using an automated video tracking system (EthoVision XT 15, Noldus Information Technology, Wageningen, Netherlands).

#### 2.5.3 Forced swim test (FST)

The FST was used to measure behavioral despair, another key depressive-like phenotype. Rats underwent a 15-min pre-swim session 24 h prior to the formal test to induce behavioral despair. For the test session, we placed each rat individually in a transparent cylindrical tank (50 cm height, 20 cm diameter) filled with water (24 ± 1 °C) to a depth of 30 cm, such that the rat could neither touch the tank bottom nor escape. The total test duration was 6 min, with the first 2 min serving as an acclimation period. We recorded the cumulative immobility time during the final 4 min, defined as floating passively in the water with only minimal movements necessary to keep the head above the surface.

### 2.6 Molecular biological assays in the hippocampus

#### 2.6.1 Enzyme-linked immunosorbent assay (ELISA)

Hippocampal concentrations of cyclic adenosine monophosphate (cAMP, Cat. No. E-EL-0056) were quantified using commercial ELISA kits from Elabscience Biotechnology Co., Ltd. (Wuhan, China). Levels of 5-hydroxytryptamine (5-HT, Cat. No. MM-0442R1), dopamine (DA, Cat. No. MM-0355R1), and norepinephrine (NE, Cat. No. MM-0556R1) were measured with kits from Jiangsu Meimian Industrial Co., Ltd. (Jiangsu, China). All assays were performed following the manufacturers’ protocols without modification. All kits were validated for rat tissue samples, with reported intra-assay coefficients of variation (CV) < 10% and inter-assay CV < 15%. Absorbance was read at 450 nm on a Tecan Infinite M200 microplate reader (Tecan Group Ltd., Männedorf, Switzerland), and analyte concentrations were calculated from standard curves generated in parallel for each assay plate.

#### 2.6.2 Quantitative real-time polymerase chain reaction (qRT-PCR)

Total RNA was isolated from frozen hippocampal tissues using Trizon Reagent combined with an RNA extraction kit (CWBIO, Beijing, China) per the manufacturer’s instructions. RNA purity and concentration were determined spectrophotometrically with a NanoPhotometer (Implen, Munich, Germany); samples with A260/A280 ratios between 1.8 and 2.2 were deemed acceptable for downstream analysis. Complementary DNA (cDNA) was synthesized from 1 μg of total RNA using the HiScript II Q RT SuperMix for qPCR (Vazyme, Nanjing, China). Quantitative PCR was performed in triplicate using SuperStar Universal SYBR Master Mix (CWBIO, Beijing, China) on a CFX Connect™ Real-Time PCR Detection System (Bio-Rad, Hercules, CA, USA). All primer pairs were designed to span exon-exon junctions to prevent genomic DNA amplification, and amplification efficiencies (90–110%) were experimentally validated for each target. *Actb* (encoding *β*-actin) served as the endogenous reference gene, and its stable expression across all experimental groups was confirmed (one-way ANOVA, *p* > 0.05). Relative mRNA expression levels of *Bdnf* and *Ntrk2* (encoding TrkB) were calculated using the 2 < sup > −ΔΔCt</sup > method. Primer sequences are listed below: *Bdnf* (forward: 5’-CCTGGCAGGCTTTGATGAGA-3′, reverse: 5’-ACCTGGTGGAACTCAGGGT-3′); *Ntrk2* (forward: 5’-GGCCGTGAAGACGCTGA-3′, reverse: 5’-ATTTGCTGAGCGATGTGCAG-3′); and *Actb* (forward: 5’-GCCATGTACGTAGCCATCCA-3′, reverse: 5’-GAACCGCTCATTGCCGATAG-3′).

### 2.7 16S rRNA gene sequencing analysis

#### 2.7.1 DNA extraction and PCR amplification

Total bacterial genomic DNA was extracted from frozen colonic fecal pellets using the QIAamp Fast DNA Stool Mini Kit (Qiagen, Hilden, Germany) following the manufacturer’s standard protocol. DNA quality and concentration were assessed using a Qubit 2.0 Fluorometer (Thermo Fisher Scientific, Waltham, MA, USA) and 1% agarose gel electrophoresis. The V3–V4 hypervariable regions of the 16S rRNA gene were amplified using barcoded universal primers (341F, 5’-CCTAYGGGRBGCASCAG-3′, 806R, 5’-GGACTACNNGGGTATCTAAT-3′). PCR reactions were carried out with Phusion High-Fidelity PCR Master Mix (New England Biolabs, Ipswich, MA, USA) under the following cycling conditions: initial denaturation at 95 °C for 3 min, followed by 30 cycles of 95 °C for 30 s, 55 °C for 30 s, and 72 °C for 45 s, with a final extension at 72 °C for 5 min.

#### 2.7.2 Library construction and sequencing

PCR amplicons were purified using AMPure XP magnetic beads (Beckman Coulter, Brea, CA, USA) to remove primer dimers and non-specific products. Sequencing libraries were prepared with the NEBNext Ultra II DNA Library Prep Kit (New England Biolabs, Ipswich, MA, USA) according to the manufacturer’s recommendations. Paired-end sequencing (2 × 250 bp) was performed on an Illumina NovaSeq 6,000 platform (Illumina Inc., San Diego, CA, USA), generating an average of 50,000 high-quality reads per sample.

#### 2.7.3 Sequencing data preprocessing and ASV analysis

Raw paired-end sequencing reads were demultiplexed and processed using QIIME 2 (version 2023.2) (Bolyen et al., 2019). The Deblur algorithm was employed for sequence denoising, quality filtering, and chimera removal, producing amplicon sequence variants (ASVs) at single-nucleotide resolution (Amir et al., 2017). Deblur was chosen over alternative denoising methods due to its superior computational efficiency, significant parallel processing capabilities, and consistent sequence identification across independent datasets. All samples met stringent quality control criteria: Q30 quality score > 90% (corresponding to a sequencing error rate < 0.1%), effective sequence retention ratio > 70%, and Goods_coverage index > 0.997, confirming sufficient sequencing depth to capture the full microbial community diversity present in the samples.

#### 2.7.4 Taxonomic annotation and diversity analysis

Taxonomic classification of ASVs was performed against the SILVA 16S rRNA gene database (version 138.1) using a naive Bayes classifier trained specifically on the V3–V4 hypervariable region, with a 97% sequence identity confidence threshold. Alpha diversity indices (Observed_ASV, Shannon, Simpson, Chao1, ACE, Goods_coverage, and PD_whole_tree) were calculated within QIIME 2. Between-group differences in alpha diversity were tested using the Wilcoxon rank-sum test with Benjamini-Hochberg false discovery rate (FDR) correction for multiple comparisons. Beta diversity was assessed using Bray–Curtis dissimilarity, weighted UniFrac, and unweighted UniFrac distances. Principal Coordinate Analysis (PCoA) was used to visualize global differences in microbial community structure between experimental groups. Statistical significance of between-group compositional differences was determined using permutational multivariate analysis of variance (PERMANOVA) with 999 permutations for each distance matrix. Unweighted Pair-group Method with Arithmetic Mean (UPGMA) clustering was performed to evaluate hierarchical similarity between samples based on phylogenetic distances.

#### 2.7.5 Differential taxa analysis and functional prediction

Linear discriminant analysis Effect Size (LEfSe) was used to identify differentially abundant taxa at all taxonomic levels between groups, with a significance threshold set at LDA score > 3.0. Predictive functional profiling of the gut microbial communities was conducted using Tax4Fun2 (version 1.1.5). Representative ASV sequences were aligned to the Ref99NR database at 97% sequence identity, and functional predictions were generated after normalization by 16S rRNA gene copy number. The average fraction of taxa unused (FTU) was 0.12, indicating high prediction reliability. KEGG Orthology (KO) annotations at Level 3 were compared between groups using the Wilcoxon rank-sum test with Benjamini-Hochberg FDR correction. Pathways with an adjusted *q*-value < 0.05 were considered significantly altered.

### 2.8 Serum untargeted metabolomics analysis based on UPLC-MS/MS

#### 2.8.1 Serum sample preparation

Serum samples were thawed on ice and vortexed briefly (10 s) to ensure homogeneity. A 50 μL aliquot of each sample was transferred to a microcentrifuge tube, and 300 μL of pre-cooled 20% acetonitrile-methanol solution containing 2-chloro-L-phenylalanine (4 μg/mL, Sigma-Aldrich, St. Louis, MO, USA) as an internal standard was added. The mixture was vortexed vigorously for 3 min and then centrifuged at 12,000 rpm for 10 min at 4 °C. A 200 μL aliquot of the supernatant was transferred to a new tube and incubated at −20 °C for 30 min to complete protein precipitation. Following a second centrifugation step (12,000 rpm, 3 min, 4 °C), 180 μL of the clear supernatant was transferred to an autosampler vial insert for UPLC-MS/MS analysis. Quality control (QC) samples were prepared by pooling equal volumes of supernatant from all experimental samples. One QC sample was injected after every 10 experimental samples throughout the analytical run to monitor instrument stability and experimental reproducibility.

#### 2.8.2 UPLC-MS/MS analytical conditions

Chromatographic separation was performed on a Thermo Vanquish UPLC system coupled to a Q Exactive HF-X high-resolution mass spectrometer (Thermo Fisher Scientific, Waltham, MA, USA) equipped with an electrospray ionization (ESI) source operating in both positive and negative ion modes. Separation was achieved on a Waters ACQUITY Premier HSS T3 Column (1.8 μm, 2.1 mm × 100 mm, Waters Corporation, Milford, MA, USA) maintained at 40 °C, with a flow rate of 0.4 mL/min and an injection volume of 4 μL. The mobile phase consisted of 0.1% (v/v) formic acid in water (A) and 0.1% (v/v) formic acid in acetonitrile (B). The gradient elution program is detailed in Table 2, and mass spectrometry operating parameters are summarized in Table 3.

**Table 2:** Gradient elution program for UPLC-MS/MS analysis.

Time (min)	Mobile phase A (%)	Mobile phase B (%)
0.0	95	5
2.0	80	20
5.0	40	60
6.0	1	99
7.5	1	99
7.6	95	5
10.0	95	5

**Table 3:** Mass spectrometry operating parameters in positive and negative ion mode.

Parameter	Positive ion mode	Negative ion mode
Spray Voltage (V)	3,500	3,200
Sheath gas (Arb)	30	30
Aux gas (Arb)	5	5
Ion transfer tube temp (°C)	320	320
Vaporizer temp (°C)	300	300
MS1 scan range (m/z)	75–1,000	75–1,000
MS1 resolution	35,000	35,000
MS1 AGC target	1.0 × 10^6^	1.0 × 10^6^
MS2 scan range (m/z)	75–1,000	75–1,000
MS2 resolution	17,500	17,500
MS2 AGC target	2.0 × 10^5^	2.0 × 10^5^
Collision energy (eV)	30, 40, 50	30, 40, 50
Dynamic exclusion (s)	3	3
Signal intensity threshold	1.0 × 10^6^ cps	1.0 × 10^6^ cps
Top N ions for MS2	10	10

#### 2.8.3 Data preprocessing and metabolite identification

Raw MS data files were converted to mzML format using ProteoWizard (version 3.0). Peak detection, retention time correction, and peak alignment were performed using the *XCMS* package in R (version 4.5.3). Data preprocessing proceeded in three sequential steps: (1) filtering, where ions with a missing rate > 50% in any experimental group were removed; (2) missing value imputation, where ions with a missing rate < 50% were imputed using the k-nearest neighbors (KNN) algorithm, and any remaining missing values were replaced with one-fifth of the minimum detected value for that ion; and (3) normalization, where peak areas were corrected using support vector regression (SVR) to minimize systematic variation. Metabolite identification was performed by matching accurate masses (mass error < 5 ppm), MS/MS fragmentation spectra, and retention times against a comprehensive database including our laboratory’s in-house reference library, public databases (HMDB, Metlin, KEGG), and predictive metabolite databases. Reliable metabolites were selected based on three criteria: a comprehensive identification score > 0.5, a coefficient of variation (CV) < 0.3 across all QC samples, and the exclusion of Level 4 metabolites (identified only by MS1 accurate mass and retention time without MS/MS fragmentation confirmation). Metabolites detected in both positive and negative ion modes were merged, retaining the entry with the highest identification level and lowest CV value for subsequent analysis. A targeted retrospective search was also conducted to identify prototype components of mCLMD in the serum metabolome based on database matching and existing literature.

#### 2.8.4 Statistical analysis and differential metabolite screening

Statistical analyses were performed using R (version 4.5.3) and the *MetaboAnalystR* package (version 3.2). Unsupervised principal component analysis (PCA) was used to visualize overall metabolic profile distribution and intragroup variation. Supervised orthogonal partial least squares discriminant analysis (OPLS-DA) was applied to maximize intergroup differences, and model validity was confirmed using 200-fold permutation tests to rule out overfitting. Differential metabolites were identified based on two criteria: a Variable Importance in Projection (VIP) score > 1 from the OPLS-DA model, and an adjusted *p*-value < 0.05 (Wilcoxon rank-sum test with Benjamini-Hochberg FDR correction). KEGG pathway enrichment analysis was performed on the identified differential metabolites, with an adjusted *p*-value < 0.05 considered statistically significant.

### 2.9 Statistical analysis

All data are presented as mean ± standard deviation (SD) unless otherwise specified. Statistical analyses were performed using R (version 4.5.3, R Foundation for Statistical Computing, Vienna, Austria) and GraphPad Prism (version 9.5.1, GraphPad Software Inc., San Diego, CA, USA). Data normality was assessed using the Shapiro–Wilk test, and homogeneity of variance was evaluated using Levene’s test. Normally distributed data with equal variance were analyzed using one-way analysis of variance (ANOVA), followed by Dunnett’s *post-hoc* test for pairwise comparisons against the model (MOD) group. For non-normally distributed data or data with unequal variance, the Kruskal–Wallis test was used, followed by Dunn’s *post-hoc* test for comparisons against the MOD group. Correlation analyses were performed using the Spearman rank correlation test. Outliers were detected using Grubbs’ test and excluded from analysis if *p* < 0.05. All statistical tests were two-tailed, and a *p*-value < 0.05 was considered statistically significant.

## 3 Results

### 3.1 mCLMD alleviates CUMS-induced depressive-like behaviors

**Figure 1:**
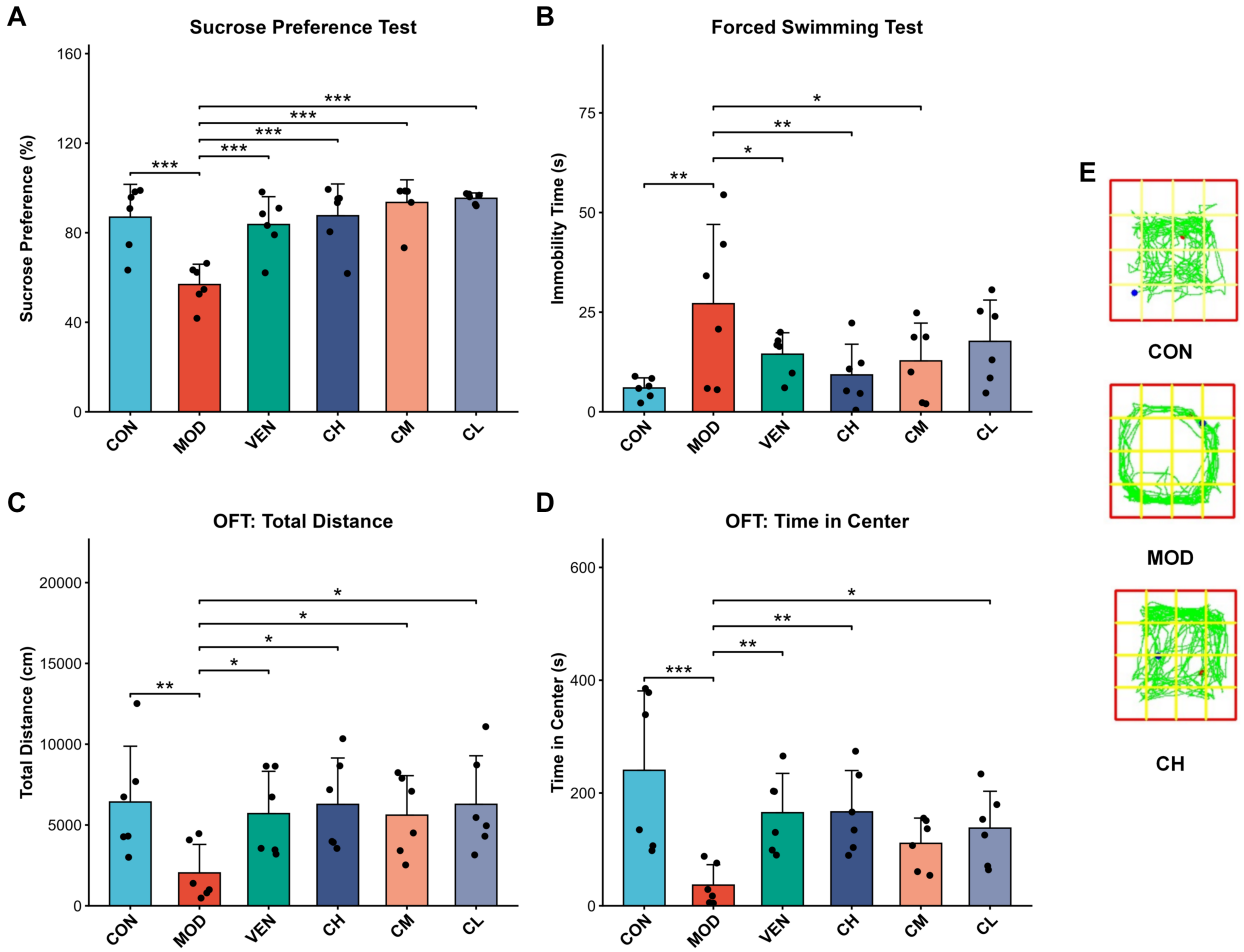
mCLMD alleviates CUMS-induced depressive-like behaviors in rats. **(A)** Sucrose preference in the Sucrose Preference Test (SPT). **(B)** Immobility time in the Forced Swim Test (FST). **(C)** Total distance traveled in the Open Field Test (OFT). **(D)** Time spent in the central zone in the OFT. **(E)** Representative movement trajectories in the OFT arena. Data are expressed as mean ± SD (*n* = 6 biological replicates per group). Statistical analysis: **(A)** one-way ANOVA followed by Dunnett’s *post-hoc* test; **(B–D)** Kruskal-Wallis test followed by Dunn’s *post-hoc* test. *p < 0.05, **p < 0.01, ***p < 0.001 versus MOD group.

To evaluate the antidepressant efficacy of mCLMD, a comprehensive battery of behavioral tests was conducted in a chronic unpredictable mild stress (CUMS)-induced rat model of depression. Chronic CUMS exposure induced anhedonia, the core depressive phenotype, as evidenced by a marked reduction in sucrose preference in the Model (MOD) group compared with the Control (CON) group (***p < 0.001). mCLMD treatment dose-dependently reversed this anhedonic deficit. All three mCLMD-treated groups (low-dose [CL], medium-dose [CM], and high-dose [CH]) exhibited significantly higher sucrose preference than the MOD group (all ***p < 0.001, Figure 1A), with efficacy comparable to that of the venlafaxine (VEN) positive control group.

Consistent with the amelioration of anhedonia, mCLMD also attenuated behavioral despair in the forced swim test (FST). CUMS exposure markedly prolonged immobility time, whereas mCLMD treatment reduced this parameter in a dose-dependent manner. Specifically, the CH and CM groups showed significantly shorter immobility times relative to the MOD group (**p < 0.01 and *p < 0.05, respectively), while no statistically significant difference was observed between the CL and MOD groups (Figure 1B).

Locomotor activity and anxiety-like behavior were next assessed using the open-field test (OFT). MOD rats exhibited a significant reduction in total distance traveled (**p < 0.01) and spent significantly less time in the central zone (***p < 0.001) compared with CON rats. mCLMD treatment restored locomotor activity across all tested doses (*p < 0.05 vs. MOD, Figure 1C). With respect to time spent in the central zone, both the CH (**p < 0.01) and CL (*p < 0.05) groups showed significant increases relative to the MOD group (Figure 1D). These behavioral changes were corroborated by representative movement trajectories: MOD rats displayed typical thigmotaxis, remaining predominantly along the arena walls, whereas CON and CH rats frequently entered and extensively explored the central region (Figure 1E). Taken together, these results demonstrate that mCLMD effectively ameliorates CUMS-induced anhedonia, behavioral despair, and anxiety-like behavior in rats.

### 3.2 Network pharmacology analysis uncovers multi-target synaptic and neurotrophic mechanisms of mCLMD

**Figure 2:**
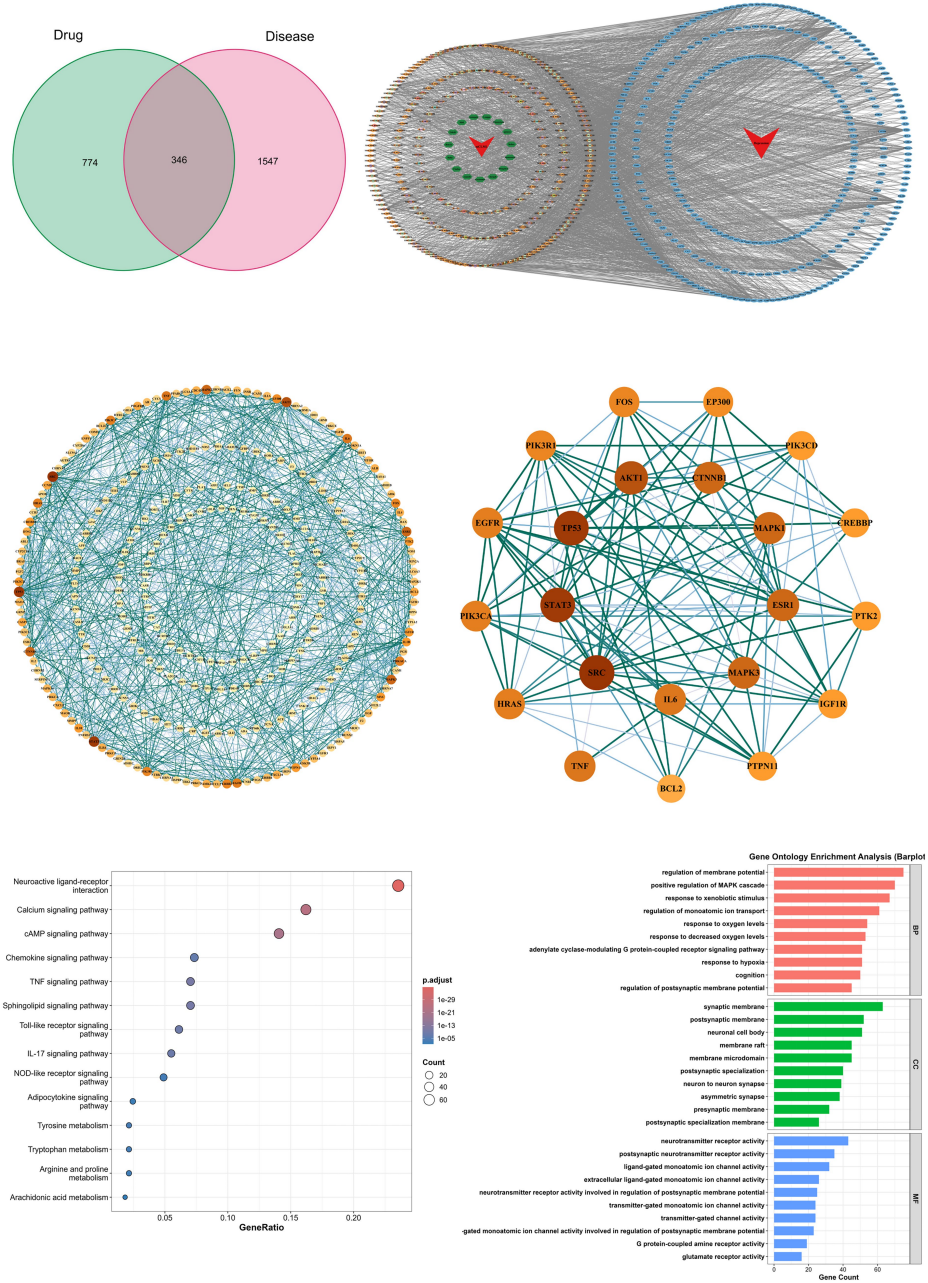
Network pharmacology predicts multi-target antidepressant mechanisms of mCLMD. **(A)** Venn diagram showing overlapping targets between mCLMD active compounds and depression-related genes. **(B)** Herb-active ingredient-target network of mCLMD. **(C)** Protein–protein interaction (PPI) network of overlapping targets (STRING database, confidence score > 0.9). **(D)** Core hub gene subnetwork screened by two rounds of median topological filtering using CytoNCA. **(E)** Bubble chart of top 20 enriched KEGG pathways for core hub targets. **(F)** Bar plot of Gene Ontology (GO) enrichment analysis, including biological process (BP), cellular component (CC), and molecular function (MF). Statistical significance for enrichment analysis was set at adjusted *p* < 0.05 (Benjamini-Hochberg correction).

To elucidate the molecular mechanisms underlying the antidepressant effects of mCLMD, an integrated network pharmacology approach was employed. Putative active compounds of mCLMD and their corresponding targets were retrieved from the TCMSP and HERB databases, while depression-associated targets were compiled from multiple well-curated disease databases. Venn diagram analysis identified 346 overlapping genes, which were designated as potential therapeutic targets of mCLMD for depression (Figure 2A).

An herb-compound-target network was then constructed to visualize the multi-component synergistic effects of the formula (Figure 2B). A protein–protein interaction (PPI) network was subsequently generated using the STRING database with a high confidence threshold of 0.90 (Figure 2C). Topological analysis of the PPI network identified a core subnetwork consisting of 22 hub genes, among which *BDNF* and *NTRK2* (encoding TrkB) are well-established key regulators of depression pathogenesis (Figure 2D).

Functional enrichment analysis was performed to explore the systemic mechanisms of mCLMD and guide experimental validation. Gene Ontology (GO) enrichment analysis (Figure 2F) revealed that the target genes were significantly enriched in biological processes and cellular components critical for central nervous system function, including regulation of membrane potential, synaptic transmission, and postsynaptic specialization. These findings strongly support the hypothesis that mCLMD exerts its effects by modulating neuroplasticity.

Importantly, Kyoto Encyclopedia of Genes and Genomes (KEGG) pathway analysis (Figure 2E) further revealed a highly coordinated “peripheral-central” dual-targeting profile. Centrally, target genes were significantly enriched in pathways directly related to neurotrophic regulation, including the cAMP signaling pathway, neuroactive ligand-receptor interaction, and serotonergic synapse. Peripherally, a prominent cluster of pathways involved in immunometabolic homeostasis was observed, encompassing systemic inflammatory cascades (TNF, IL-17, Toll-like receptor, and NOD-like receptor signaling pathways) and key metabolic networks (tryptophan metabolism, sphingolipid signaling, and arachidonic acid metabolism).

Taken together, these bioinformatic results suggest that mCLMD exerts its antidepressant effects not solely through direct binding to central nervous system receptors. Instead, it acts via a “dual-drive” mechanism that integrates central synaptic modulation with systemic regulation of the peripheral immunometabolic microenvironment. This mechanistic hypothesis provided the rationale for the comprehensive *in vivo* experimental design, encompassing 16S rRNA gut microbiome sequencing, untargeted serum metabolomics, and molecular validation of the hippocampal cAMP/BDNF/TrkB signaling pathway.

### 3.3 mCLMD restores hippocampal monoamine neurotransmitters and upregulates cAMP/BDNF/TrkB pathway transcription

**Figure 3:**
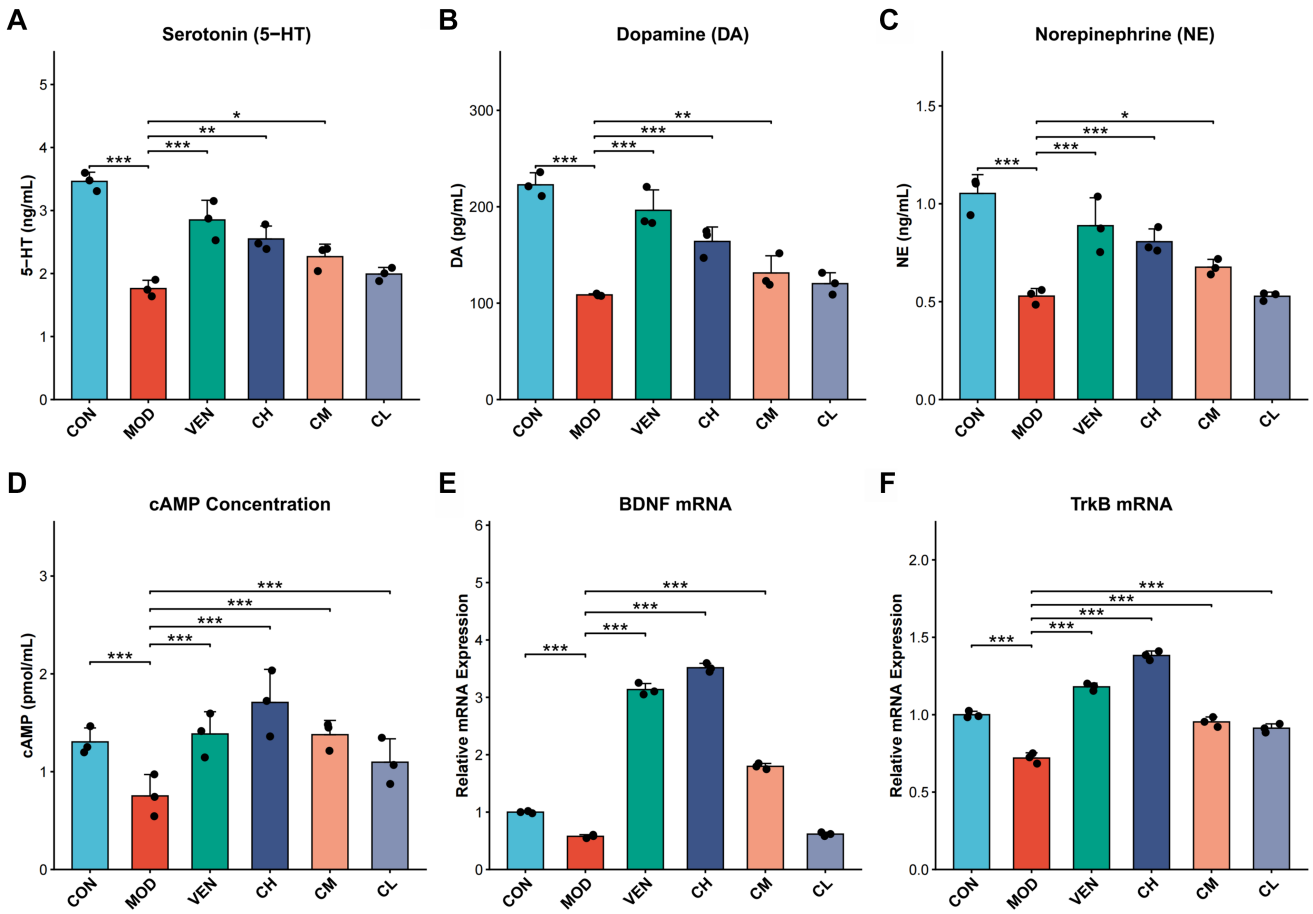
mCLMD restores hippocampal monoaminergic neurotransmission and activates the cAMP-BDNF–TrkB signaling pathway in CUMS rats. **(A–C)** Hippocampal concentrations of 5-hydroxytryptamine (5-HT, **A**), dopamine (DA, **B**), and norepinephrine (NE, **C**) measured by ELISA. **(D)** Hippocampal cAMP concentration determined by ELISA. **(E,F)** Relative mRNA expression levels of *Bdnf* **(E)** and *Ntrk2* (encoding TrkB, **F**) assessed by quantitative real-time PCR (qRT-PCR), with *β*-actin as the internal reference. Data are expressed as mean ± SD with individual data points overlaid (*n* = 3 biological replicates per group). All data were normally distributed with equal variance and analyzed via one-way ANOVA followed by Dunnett’s *post-hoc* test. *p < 0.05, **p < 0.01, ***p < 0.001 versus MOD group.

Building on the network pharmacology findings that identified synaptic transmission and the cAMP signaling pathway as core therapeutic modules, the effects of mCLMD on hippocampal neurochemistry were next examined. ELISA assays revealed that CUMS exposure caused a marked reduction in hippocampal levels of 5-hydroxytryptamine (5-HT), dopamine (DA), and norepinephrine (NE) (all ***p < 0.001 vs. CON). High-dose mCLMD (CH) treatment completely restored all three monoamine neurotransmitters (5-HT: **p < 0.01; DA and NE: ***p < 0.001 vs. MOD, Figures 3A–C). Medium-dose mCLMD (CM) also significantly increased DA levels (**p < 0.01) and induced moderate increases in 5-HT and NE concentrations (*p < 0.05 vs. MOD).

Key components of the cAMP/BDNF/TrkB signaling axis were then evaluated. CUMS significantly suppressed hippocampal cAMP levels (***p < 0.001 vs. CON). mCLMD treatment reversed this suppression in a dose-dependent manner, with all three doses producing significant elevations in cAMP concentrations (all ***p < 0.001 vs. MOD, Figure 3D). At the transcriptional level, qPCR analysis confirmed that CUMS significantly downregulated mRNA levels of both *Bdnf* and its receptor *Ntrk2* (encoding TrkB). All three doses of mCLMD significantly increased *Ntrk2* mRNA expression (all ***p < 0.001 vs. MOD, Figure 3F), while *Bdnf* mRNA levels were significantly upregulated in both the high-dose and medium-dose groups (***p < 0.001 vs. MOD, Figure 3E).

The magnitude of these restorative changes in the CH group was comparable to that observed in the venlafaxine (VEN) positive control group. Collectively, these data demonstrate that mCLMD normalizes hippocampal monoamine neurotransmitter levels and activates the cAMP/BDNF/TrkB signaling pathway at the transcriptional level.

### 3.4 mCLMD restructures the gut microbial community disrupted by chronic stress

#### 3.4.1 mCLMD reverses CUMS-induced alterations in gut microbial diversity

**Figure 4:**
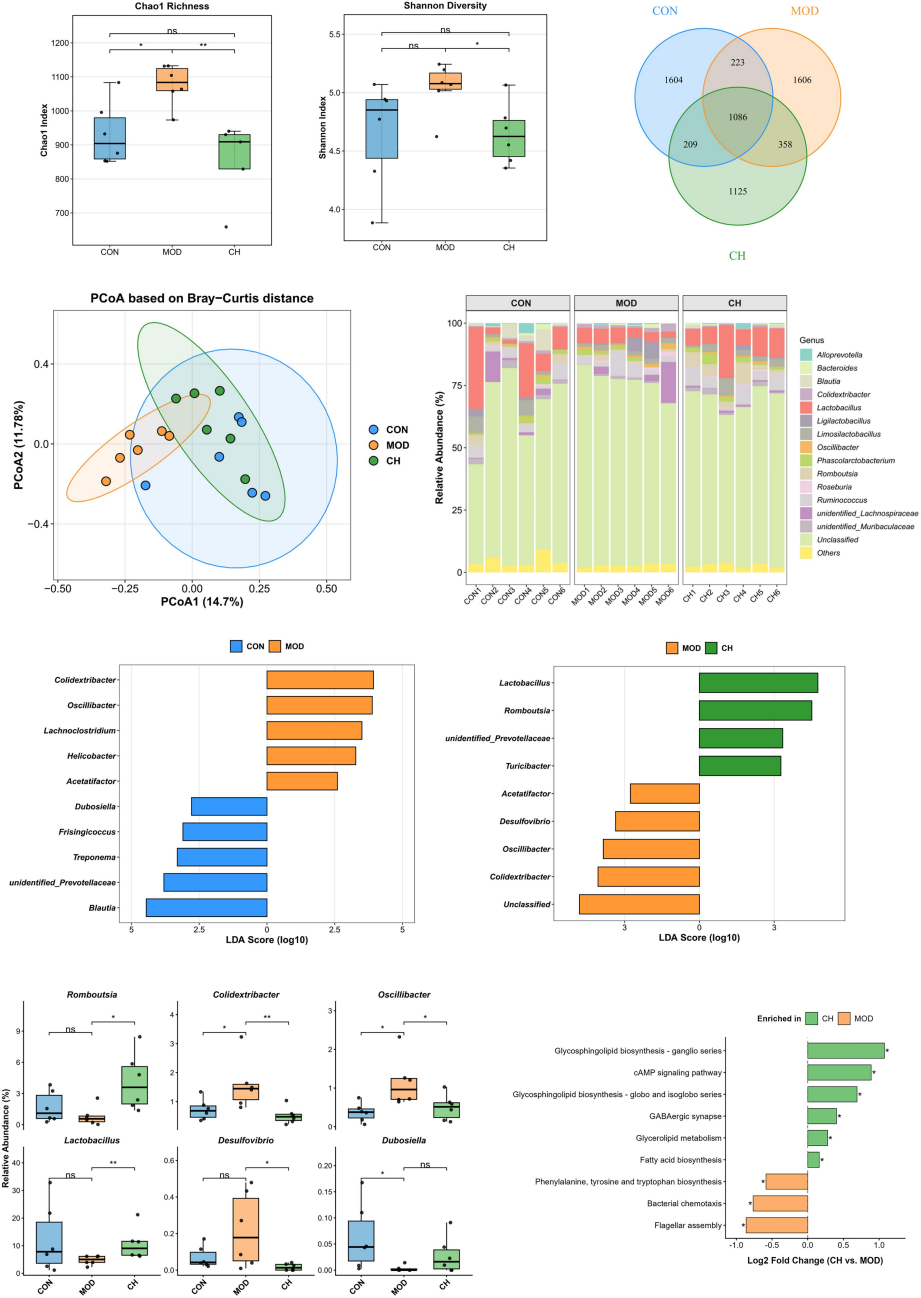
High-dose mCLMD (CH) reverses CUMS-induced gut microbial dysbiosis and normalizes predictive microbial functions in rats. **(A,B)** Alpha diversity indices: Chao1 richness **(A)** and Shannon diversity **(B)**. **(C)** Venn diagram showing shared and unique amplicon sequence variants (ASVs) among groups. **(D)** Principal Coordinate Analysis (PCoA) based on Bray–Curtis dissimilarity, showing overall microbial community structure differences (PERMANOVA, *p* < 0.01). **(E)** Stacked bar plot of relative abundance at the genus level. **(F,G)** Linear discriminant analysis Effect Size (LEfSe) identifying differential microbial biomarkers between CON vs. MOD **(F)** and MOD vs. CH **(G)** (LDA score > 3.0, *p* < 0.05). **(H)** Box plots showing relative abundance of core depression-related genera. **(I)** Tax4Fun2 predictive functional profiling showing significantly altered KEGG Level 3 pathways between MOD and CH groups. Data are visualized as box-and-whisker plots with individual data points overlaid (*n* = 6 biological replicates per group). Statistical analysis: **(A,B, H)** Kruskal-Wallis test followed by Dunn’s *post-hoc* test; **(D)** PERMANOVA with 999 permutations; **(I)** Wilcoxon rank-sum test with Benjamini-Hochberg FDR correction. * *p* < 0.05, ** *p* < 0.01, ns: not significant.

Alpha diversity indices were compared between groups using the Wilcoxon rank-sum test. The Chao1 index, a measure of microbial richness, was significantly higher in the MOD group than in the CON group (*p < 0.05; Figure 4A)—a finding consistent with previous clinical and preclinical studies showing increased gut microbial richness in both patients with major depressive disorder (MDD) and chronic stress-induced depression animal models (Duan et al., 2021). High-dose mCLMD completely reversed this increase, restoring microbial richness to levels indistinguishable from the CON group (**p < 0.01 vs. MOD). The Shannon diversity index, which reflects both richness and evenness, showed no significant difference between the CON and MOD groups, but was significantly lower in the CH group than in the MOD group (*p < 0.05; Figure 4B), indicating that mCLMD reshaped the community structure by selectively reducing the abundance of potentially pathogenic taxa.

A Venn diagram showed the distribution of shared and unique amplicon sequence variants (ASVs) across the three groups: 1086 ASVs were common to all groups, while 1,604, 1,606, and 1,125 unique ASVs were identified in the CON, MOD, and CH groups, respectively (Figure 4C). Beta diversity analysis using Principal Coordinate Analysis (PCoA) based on Bray–Curtis distances revealed distinct separation of microbial community structures across the three groups (PERMANOVA, **p < 0.01), with PCoA1 and PCoA2 explaining 14.7 and 11.78% of the total variance, respectively (Figure 4D). The CH group clustered significantly closer to the CON group than to the MOD group, confirming that mCLMD partially restored the overall microbial community structure disrupted by chronic stress.

#### 3.4.2 mCLMD normalizes depression-associated microbial Dysbiosis and restores predictive metabolic functions

At the phylum level, *Bacillota* and *Bacteroidota* were the dominant taxa across all groups, consistent with the normal gut microbial composition of rats. At the genus level, CUMS exposure caused pronounced shifts in the abundance of multiple taxa closely associated with depression pathogenesis, and these changes were largely reversed by mCLMD treatment (Figure 4E).

Linear discriminant analysis Effect Size (LEfSe) with an LDA score threshold of 3.0 was used to identify key differential taxa driving community separation. In the CON vs. MOD comparison (Figure 4F), the MOD group was characterized by significant enrichment of pro-inflammatory genera associated with depression, including *Colidextribacter* (LDA = 3.93), *Oscillibacter* (LDA = 3.88), and *Desulfovibrio* (LDA = 3.60). In contrast, *Blautia* (LDA = 4.46)—a genus with well-documented anti-inflammatory and antidepressant-like effects in preclinical models—was significantly depleted in the MOD group. In the MOD vs. CH comparison (Figure 4G), mCLMD treatment led to a sharp decline in these pro-inflammatory genera, while significantly enriching beneficial taxa such as *Lactobacillus* (LDA = 4.75) and *Romboutsia* (LDA = 4.51).

The relative abundances of these key taxa were further quantified to confirm the LEfSe findings (Figure 4H). *Colidextribacter*, *Oscillibacter*, and *Desulfovibrio* were all significantly enriched in the MOD group and reduced to levels comparable to the CON group by mCLMD treatment (all *p < 0.05). Among beneficial genera, *Lactobacillus* and *Romboutsia* were significantly increased in the CH group compared with the MOD group (both *p < 0.05), while *Dubosiella*—a taxon markedly depleted by chronic stress—showed a clear trend toward recovery after mCLMD treatment. Notably, the restorative effects of mCLMD on gut microbial dysbiosis were more pronounced than those of venlafaxine, which only exhibited mild trends toward normalization of gut microbial profiles.

To explore the potential functional consequences of these mCLMD-induced microbial shifts, predictive functional profiling was performed using Tax4Fun2. Comparative analysis of KEGG Level 3 pathways between the MOD and CH groups revealed significant inferred functional reprogramming of the gut microbiome (Figure 4I). Specifically, the CH group showed significant depletion of stress-associated pathways enriched in the MOD group, including pro-inflammatory pathways such as bacterial chemotaxis (*q* = 0.018) and flagellar assembly (*q* = 0.016), as well as phenylalanine, tyrosine, and tryptophan biosynthesis (*q* = 0.018). Conversely, mCLMD treatment resulted in a predicted enrichment of functional modules critical for neuroprotection and metabolic homeostasis that were suppressed by CUMS, including the cAMP signaling pathway (*q* = 0.041), GABAergic synapse (*q* = 0.014), fatty acid biosynthesis (*q* = 0.044), and glycerolipid metabolism (*q* = 0.018). It should be emphasized that these are computational inferences derived from 16S rRNA gene profiles and do not represent direct metagenomic or transcriptomic evidence.

These predictive functional changes are highly consistent with both the serum metabolomics findings and the targeted molecular validation of hippocampal neurotrophic signaling, further supporting the hypothesis that mCLMD exerts its antidepressant effects, at least in part, through modulation of the microbiota-lipid-brain axis.

### 3.5 mCLMD reprograms the serum metabolome and normalizes CUMS-induced metabolic disturbances

#### 3.5.1 mCLMD reverses global metabolic dysregulation induced by chronic stress

**Figure 5:**
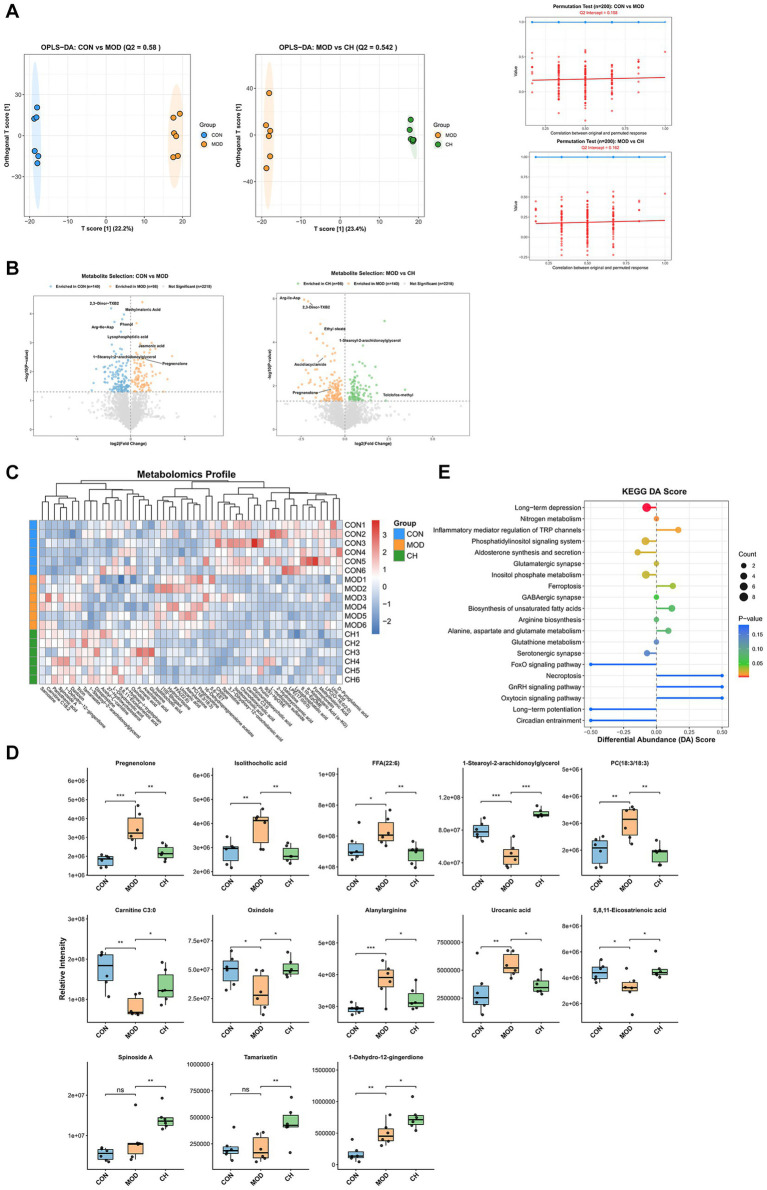
mCLMD reprograms serum metabolome and normalizes CUMS-induced metabolic disturbances. **(A)** Orthogonal Partial Least Squares Discriminant Analysis (OPLS-DA) score plots with corresponding 200-permutation test results (insets), showing metabolic separation between CON vs. MOD and MOD vs. CH groups. **(B)** Volcano plots illustrating differentially abundant metabolites in CON vs. MOD (left) and MOD vs. CH (right) comparisons. **(C)** Hierarchical clustering heatmap of the top 50 biologically relevant differential metabolites (Z-score normalized). **(D)** Violin plots with overlaid box plots showing relative abundances (Z-score normalized) of representative core metabolic biomarkers. **(E)** Lollipop plot of Differential Abundance (DA) scores showing core KEGG metabolic pathways modulated by high-dose mCLMD (CH vs. MOD). Data are from *n* = 6 biological replicates per group.Statistical analysis: **(B)** Student’s *t*-test with Benjamini-Hochberg FDR correction; **(D)** Kruskal-Wallis test followed by Dunn’s *post-hoc* test; **(E)** Fisher’s exact test with Benjamini-Hochberg FDR correction. Significantly differential metabolites were defined as Variable Importance in Projection (VIP) > 1.0 and adjusted *p* < 0.05. The asterisks indicate statistical significance compared to the MOD group: * *p* < 0.05, ** *p* < 0.01, *** *p* < 0.001.

Orthogonal partial least squares discriminant analysis (OPLS-DA) revealed distinct separation of serum metabolic profiles across the CON, MOD, and CH groups (Figure 5A), indicating that CUMS exposure induced profound systemic metabolic disturbances that were largely reversed by high-dose mCLMD treatment. Model validity was verified via 200-fold permutation tests, showing no signs of overfitting (CON vs. MOD: *Q*^2^ = 0.58; MOD vs. CH: *Q*^2^ = 0.542).

Volcano plot analysis identified 238 significantly differential metabolites in the CON vs. MOD comparison and 238 differential metabolites in the MOD vs. CH comparison (Figure 5B). Among the metabolites significantly altered by CUMS, 78.3% were normalized by high-dose mCLMD (defined as metabolites showing a significant difference between the MOD and CON groups, but no significant difference between the CH and CON groups post-treatment). Hierarchical clustering analysis of the top 50 biologically relevant differential metabolites (variable importance in projection [VIP] > 1, adjusted *p* < 0.05) confirmed that the stress-induced metabolic dysregulation pattern was effectively reversed in the CH group (Figure 5C). These differential metabolites were predominantly involved in three core biological processes: lipid homeostasis, amino acid metabolism, and bile acid biosynthesis—all closely linked to the microbiota-gut-brain axis.

#### 3.5.2 mCLMD normalizes key metabolic biomarkers of depression

Between-group comparisons of individual metabolite levels were performed using the Wilcoxon rank-sum test. Representative endogenous metabolites and exogenous phytochemicals were selected for targeted quantitative validation based on their contribution to group separation and biological relevance to depression pathogenesis (Figure 5D).

CUMS exposure significantly increased pregnenolone levels (*p < 0.01 vs. CON), a core biomarker of neurosteroid network dysregulation and impaired systemic stress response in both clinical depression and preclinical stress models.(Cavaleri et al., 2026) High-dose mCLMD treatment reversed this abnormal accumulation (*p < 0.01 vs. MOD). CUMS also caused severe depletion of serum 1-stearoyl-2-arachidonoylglycerol (SAG) (*p < 0.01 vs. CON), a critical intracellular lipid second messenger and direct precursor of the neuroprotective endocannabinoid 2-arachidonoylglycerol (2-AG), which plays a well-established role in buffering stress and resolving neuroinflammation.(Zhang et al., 2026) mCLMD treatment restored SAG to normal levels (*p < 0.01 vs. MOD), indicating the reactivation of neuroprotective lipid signaling. mCLMD also restored stress-depleted levels of short-chain acylcarnitine C3:0 (*p* < 0.05 vs. MOD), a key regulator of mitochondrial fatty acid oxidation. The formula exerted targeted modulatory effects on broader lipid pathways, significantly downregulating specific bile acids (e.g., isolithocholic acid) and free fatty acids (e.g., FFA 22:6) compared with the model group (both *p* < 0.05), reflecting comprehensive systemic lipid reprogramming.

In amino acid and purine metabolism, mCLMD attenuated CUMS-induced pathological alterations. Stress-elevated urocanic acid levels were normalized following mCLMD administration (*p < 0.01 vs. MOD). Dysregulated metabolites closely linked to systemic neuroinflammation and synaptic function, such as alanylarginine and oxindole, were partially reversed and showed clear recovery trends.

Through the targeted retrospective search described in Section 2.8.3, multiple intact bioactive phytochemicals derived from mCLMD (including spinoside A, tamarixetin, and 1-dehydro-12-gingerdione) were successfully detected and significantly enriched in the systemic circulation of treated rats (*p* < 0.05 vs. MOD). This confirms the *in vivo* bioavailability of the formula. These circulating phytochemicals possess the potential to cross the blood–brain barrier (BBB) and exert direct neuroprotective effects—as suggested by previous reports on the brain permeability of essential oil constituents and specific flavonoids—although their presence within the central nervous system was not directly quantified in the current study (Table 4) (Ma et al., 2012; Zhao et al., 2015).

**Table 4:** Identification of putative circulating phytochemicals derived from mCLMD in rat serum.

Metabolite	Mode	RT (min)	m/z	Formula	Corrected putative herb source (Latin name)
Tamarixetin	POS	1.9248	339.04	C16H12O7	*Semen Ziziphi Spinosae* / *Radix Scutellariae*
Spinoside A	NEG	6.5505	697.358	C39H56O12	*Semen Ziziphi Spinosae*
1-Dehydro-12-gingerdione	NEG	8.6516	433.261	C23H34O4	*Rhizoma Pinelliae Praeparatum*
[8]-Gingerol	NEG	7.498	321.212	C19H30O4	*Rhizoma Pinelliae Praeparatum*
[10]-Shogaol	NEG	6.9586	331.229	C21H32O3	*Rhizoma Pinelliae Praeparatum*
Glycyrrhizin	NEG	8.8211	821.41	C42H62O16	*Radix Glycyrrhizae*
Liquiritin	NEG	3.9517	417.121	C21H22O9	*Radix Glycyrrhizae*
Atractylenolide III	NEG	5.7278	247.135	C15H20O3	*Radix Codonopsis*
Pachymic acid	POS	6.931	511.391	C33H52O5	*Sclerotium Poriae Pararadicis*
Cinnamaldehyde	POS	6.5518	115.054	C9H8O	*Ramulus Cinnamomi*
Coumarin	POS	8.8642	111.021	C9H6O2	*Ramulus Cinnamomi*
4-Hydroxycinnamic acid	POS	1.2891	165.054	C9H8O3	*Rhizoma Chuanxiong* / *Ramulus Cinnamomi*
Senkyunolide N	NEG	4.9279	249.109	C12H18O4	*Rhizoma Chuanxiong*
Dihydroferulic acid	NEG	4.47	195.069	C10H12O4	*Rhizoma Chuanxiong*/*Fructus Tritici Levis*
Apigenin 7-glucuronide	NEG	3.6999	445.078	C21H18O11	*Fructus Tritici Levis*

#### 3.5.3 mCLMD modulates key metabolic pathways linked to depression pathogenesis

To identify the core metabolic cascades directionally modulated by high-dose mCLMD, KEGG pathway enrichment analysis was performed using differential abundance (DA) scores (Figure 5E). The DA score integrates both the magnitude of individual metabolite changes and their statistical significance, providing a quantitative measure of overall pathway activation or inhibition.

Pathways with negative DA scores were hyperactivated in the CUMS-exposed MOD group and normalized by mCLMD. These included long-term depression (*p* = 0.002, DA = −0.074) and the phosphatidylinositol signaling system (*p* = 0.021, DA = −0.083), both closely linked to synaptic dysfunction and neuroinflammation in depression. Pathways with positive DA scores were suppressed by chronic stress and upregulated after mCLMD administration. These included inflammatory mediator regulation of TRP channels (*p* = 0.014, DA = 0.167) and biosynthesis of unsaturated fatty acids (*p* = 0.071, DA = 0.118), which are critical for maintaining lipid homeostasis and neuroprotective signaling.

These findings demonstrate that mCLMD extensively reprograms CUMS-induced peripheral metabolic disturbances and exerts targeted modulatory effects on pathways linked to neuroinflammation, synaptic plasticity, and lipid homeostasis. These metabolic changes align with the observed alterations in gut microbial composition and hippocampal neurotrophic signaling, supporting the link between the peripheral regulatory actions of mCLMD and its central antidepressant effects.

### 3.6 Integrated multi-omics analysis reveals key microbiota-metabolite associations correlated with mCLMD’s antidepressant-like effects

#### 3.6.1 Global omics concordance

**Figure 6:**
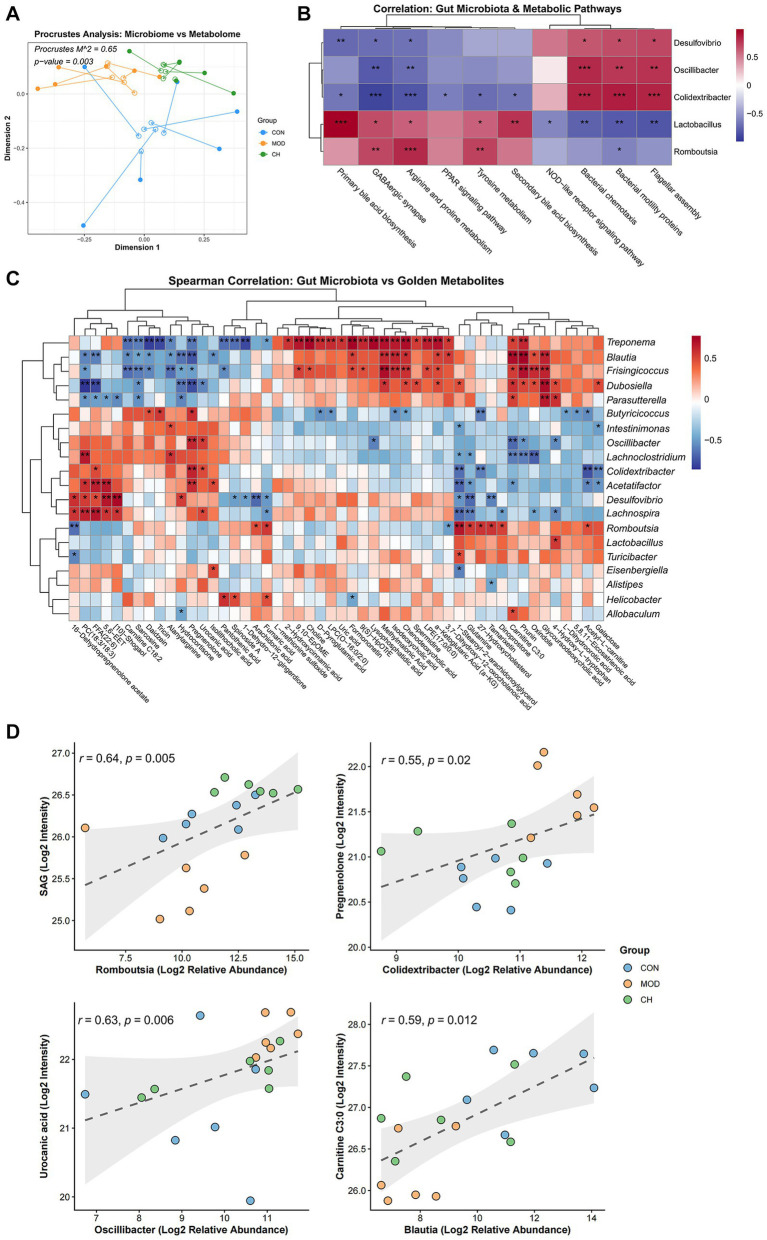
Integrated multi-omics analysis reveals microbiota-metabolite interplay associated with mCLMD’s antidepressant effects. **(A)** Procrustes analysis demonstrating significant global concordance between gut microbiome and serum metabolome profiles (*M*^2^ = 0.65, *p* = 0.003). **(B)** Spearman correlation heatmap between key differential genera and circulating metabolites. **(C)** Predictive functional pathway enrichment correlation analysis displaying a functional dichotomy between pathogenic and beneficial microbial communities. **(D)** Representative microbe-metabolite correlation scatter plots with 95% confidence intervals validating core regulatory axes: *Romboutsia* vs. 1-stearoyl-2-arachidonoylglycerol (SAG); *Colidextribacter* vs. pregnenolone; *Oscillibacter* vs. urocanic acid; and *Blautia* vs. carnitine C3:0. Statistical analysis: **(A)** Procrustes test with 999 permutations; **(B–D)** Spearman rank correlation.

To define the functional link between mCLMD-induced gut microbial remodeling and systemic metabolic normalization, integrated multi-omics correlation analyses were performed. Procrustes analysis using Bray–Curtis distances identified a highly significant global concordance between gut microbial community structure and serum metabolome profiles (*M*^2^ = 0.65, *p* = 0.003; Figure 6A). This strong bidirectional coupling confirms that mCLMD-induced systemic metabolic reprogramming is fundamentally driven by changes in the gut microbiome.

#### 3.6.2 Taxonomic-metabolic correlation networks and functional enrichment

Spearman correlation heatmaps were generated to map pairwise associations between differentially abundant microbial taxa and circulating metabolites (Figure 6B). Pro-inflammatory opportunistic pathogens enriched by CUMS, including *Oscillibacter* and *Colidextribacter*, correlated positively with stress-elevated metabolites (e.g., pregnenolone, urocanic acid) and negatively with neuroprotective energy-regulating lipids such as 1-stearoyl-2-arachidonoylglycerol (SAG) and carnitine C3:0. Beneficial genera enriched by mCLMD (*Romboutsia*, *Lactobacillus*, *Blautia*) showed the opposite pattern, correlating with the restoration of the endocannabinoid precursor pool.

Predictive pathway enrichment correlation analysis was performed to interpret these host-microbiome interactions functionally (Figure 6C). This analysis revealed distinct functional profiles between pathogenic and beneficial microbial communities. CUMS-enriched pathogenic genera (*Colidextribacter*, *Oscillibacter*, *Desulfovibrio*) correlated positively with pro-inflammatory and structural pathways associated with opportunism, including bacterial chemotaxis, flagellar assembly, and bacterial motility proteins (all *p* < 0.05). These same taxa correlated negatively with key neuroactive pathways, most notably GABAergic synapse and tyrosine metabolism—the essential precursor pathway for catecholamine neurotransmitters (*Colidextribacter* vs. GABAergic synapse: *r* = −0.909, *p* < 0.001).

Beneficial taxa enriched by mCLMD showed restorative functions. *Lactobacillus* correlated strongly with both primary (*r* = 0.993, *p* < 0.001) and secondary bile acid biosynthesis (*r* = 0.790, *p* < 0.01), identifying it as a core microbial driver of gut-liver-brain bile acid and lipid axis remodeling. Both *Lactobacillus* and *Romboutsia* correlated positively with GABAergic synapse, tyrosine metabolism, and arginine/proline metabolism (all *p* < 0.05), consistent with systemic replenishment of neurotransmitter precursors. *Lactobacillus* also correlated negatively with pro-inflammatory immune cascades, primarily the NOD-like receptor signaling pathway.

#### 3.6.3 Validation of core microbe-metabolite regulatory axes

The strongest microbe-metabolite pairs from the correlation networks—representing the core regulatory axes of mCLMD—were validated using scatter plots (Figure 6D). *Romboutsia* correlated positively with 1-stearoyl-2-arachidonoylglycerol (SAG) (Spearman *ρ* = 0.637, *p* < 0.01), confirming that mCLMD-induced expansion of this beneficial genus directly supports neuroprotective endocannabinoid lipid signaling. *Colidextribacter* correlated positively with pregnenolone (Spearman *ρ* = 0.550, *p* < 0.05), validating that suppression of this pro-inflammatory taxon alleviates systemic neurosteroid dysregulation. *Oscillibacter* also correlated positively with urocanic acid (Spearman *ρ* = 0.633, *p* < 0.01), linking this pathogen to amino acid and neuroinflammatory metabolic disturbances. *Blautia* correlated positively with carnitine C3:0 (Spearman *ρ* = 0.587, *p* < 0.05).

The integrated multi-omics analysis highlights a potential distinct microbiota-lipid-brain regulatory axis. mCLMD ameliorates depressive-like behaviors by globally reversing gut dysbiosis. This reversal subsequently normalizes peripheral neurosteroid levels (e.g., pregnenolone), restores systemic bile acid homeostasis, and increases the availability of endocannabinoid precursors (e.g., SAG) and neuroactive amino acids. These changes in circulating metabolites alleviate peripheral inflammation. This systemic shift supports central neuroplasticity and promotes the transcriptional upregulation of the hippocampal cAMP-BDNF–TrkB pathway.

## 4 Discussion

Traditional Chinese medicine (TCM) formulations exert their therapeutic effects through synergistic interactions between multiple components acting on diverse biological targets. Here, network pharmacology predicted that modified Chaihu Longgu Muli Decoction (mCLMD) produces antidepressant-like effects via both direct central receptor interactions and a systemic “peripheral-to-central” regulatory mechanism. A key challenge is identifying the specific biological pathways linking peripheral immunometabolic changes to central neuroplasticity. Our integrated multi-omics (Zhao et al., 2024) and targeted hippocampal analyses address this critical gap by suggesting that the microbiota-lipid-brain axis may play a crucial role as a potential mediator of mCLMD’s therapeutic action—a concept supported by recent pharmacological reviews of TCM for depression (Li et al., 2023).

Consistent with our *a priori* hypothesis, 16S rRNA gene sequencing identified the gut microbiota as a key peripheral target of mCLMD. At the taxonomic level, chronic unpredictable mild stress (CUMS) exposure significantly enriched pro-inflammatory, depression-associated genera including *Colidextribacter* and *Oscillibacter*. Both genera are consistently elevated across depressive-like animal models and clinical major depressive disorder (MDD) cohorts (Zhao et al., 2022; Gao et al., 2023), where they compromise intestinal barrier integrity and propagate depressive-like phenotypes through immune signaling and aberrant vagus nerve activation (Liu et al., 2023). High-dose mCLMD treatment significantly reduced the abundance of these pathobionts. Conversely, mCLMD significantly enriched beneficial genera such as *Romboutsia*, *Lactobacillus*, and *Blautia*. *Lactobacillus* species are well-characterized psychobiotics that alleviate depressive-like behaviors by reinforcing intestinal tight junctions and attenuating neuroinflammation (Dziedzic et al., 2024). These shifts indicate that mCLMD promotes an anti-inflammatory gut microbial environment, which may mediate its downstream systemic effects.

The gut microbiota influences host physiology primarily by regulating metabolism and generating bioactive mediators. Our UPLC-MS/MS-based untargeted metabolomics revealed that mCLMD largely reverses CUMS-induced systemic metabolic dysregulation, particularly within lipid and neurosteroid networks. One key observation was the normalization of pregnenolone, a core neurosteroid whose abnormal accumulation reflects severe hypothalamic–pituitary–adrenal (HPA) axis dysregulation and systemic stress (Cavaleri et al., 2026). By correcting pregnenolone levels, mCLMD alleviates peripheral neurosteroid toxicity (Cao et al., 2025; Cavaleri et al., 2026). mCLMD also markedly reversed the stress-induced depletion of 1-stearoyl-2-arachidonoylglycerol (SAG), a critical diacylglycerol and direct precursor to 2-arachidonoylglycerol (2-AG) (Zhang et al., 2026). 2-AG is the most abundant endogenous ligand of the endocannabinoid system, which plays a well-established role in buffering stress, resolving neuroinflammation, and supporting synaptic plasticity (Zhang et al., 2026). The restoration of peripheral SAG suggests a potential repair of the endocannabinoid system. However, it is important to note that this remains speculative based on circulating metabolites. Future studies should quantitatively target 2-AG and cannabinoid receptor type 1 (CB1) in brain tissues to definitively confirm this peripheral-to-central lipid signaling axis. Additionally, restoration of short-chain acylcarnitine C3:0 indicates improved mitochondrial fatty acid *β*-oxidation, further reducing peripheral metabolic burden (Hamilton et al., 2020; Liu et al., 2026).

Integrated correlation analysis further linked these taxonomic changes to the observed metabolic phenotypes. The pathobiont *Colidextribacter* is strongly correlated with pregnenolone, suggesting that its overgrowth is strongly correlated with stress-induced neurosteroid dysregulation. Conversely, *Romboutsia*, a beneficial genus enriched by mCLMD, correlated strongly positively with SAG, indicating its key role in restoring the systemic endocannabinoid precursor pool. Functional pathway analysis also highlighted *Lactobacillus* as a core driver of restored primary and secondary bile acid biosynthesis, which is essential for lipid absorption and gut-liver-brain signaling (MahmoudianDehkordi et al., 2022).

These coordinated changes in circulating metabolites systemically support central neuroplasticity. Peripheral lipid mediators, including endocannabinoid precursors and bile acids, can cross the blood–brain barrier (BBB) or signal through vagal afferents to directly modulate CNS function. Restored endocannabinoid tone and resolved neurosteroid toxicity are known to reduce hippocampal neuroinflammation, thereby relieving suppression of the cAMP-BDNF–TrkB neurotrophic pathway. Recovered BDNF signaling in turn enhances neuronal survival and synaptic plasticity, which likely underlies the observed normalization of hippocampal monoamine neurotransmitter levels (5-HT, DA, NE). The detection of intact bioactive constituents, such as spinoside A and tamarixetin, in the systemic circulation highlights additional direct pharmacological effects of mCLMD. While we did not directly validate the brain distribution of these compounds, their documented ability to penetrate the BBB suggests they may exert direct neuroprotective effects (Dajas et al., 2015; Qiao et al., 2016), acting synergistically with microbiota-dependent indirect pathways to stabilize the hippocampal microenvironment (Zhao et al., 2023).

Thus, while our study provides a systemic framework for mCLMD’s antidepressant effects, several limitations warrant consideration. First, our multi-omics approach highlights systemic associations rather than strict causality. Future mechanistic validation using fecal microbiota transplantation (FMT) or antibiotic-mediated depletion is essential. Second, due to the limited volume of dissected hippocampal tissue—which was prioritized for ELISA and qRT-PCR assays—we could not perform protein-level validations (e.g., Western blot for BDNF, p-TrkB, and p-CREB). Since mRNA changes do not necessarily reflect functional protein activation, our conclusions regarding neuroplasticity signaling are preliminary and require future protein-level confirmation. Third, the sample size (n = 6 per group) was determined based on previous exploratory multi-omics studies in CUMS rodent models, which have consistently reported significant behavioral and omics-level differences with comparable group sizes (Duan et al., 2021; Lv et al., 2021). While standard for this context, it remains relatively underpowered for high-dimensional omics, carrying a theoretical risk of overfitting. To mitigate this, we employed strict statistical penalization (e.g., FDR corrections, permutation testing). Additionally, omics profiling was restricted to the high-dose group to establish maximal molecular boundaries; subsequent studies should include dose–response multi-omics assessments in larger cohorts. Fourth, the microbial functional pathways discussed were computationally inferred (Tax4Fun2) from 16S rRNA profiles. Shotgun metagenomics is required to confirm these predictive functional estimations. Finally, reduced center time in the open-field test was interpreted as anxiety-like behavior; however, concurrent decreases in total locomotion may confound this interpretation by reflecting general motor fatigue. Future studies using specific anxiety paradigms (e.g., elevated plus maze) are needed to disentangle true anxiety from widespread locomotor deficits.

## Data Availability

The datasets presented in this study can be found in online repositories. The names of the repository/repositories and accession number(s) can be found in the article/Supplementary material.

## References

[ref1] Amir A. Mcdonald D. Navas-Molina J. A. Kopylova E. Morton J. T. Zech Xu Z. . (2017). Deblur rapidly resolves single-nucleotide community sequence patterns. mSystems 2:e00191-16. doi: 10.1128/msystems.00191-16, 28289731 PMC5340863

[ref2] Bolyen E. Rideout J. R. Dillon M. R. Bokulich N. A. Abnet C. C. Al-Ghalith G. A. . (2019). Reproducible, interactive, scalable and extensible microbiome data science using QIIME 2. Nat. Biotechnol. 37, 852–857. doi: 10.1038/s41587-019-0209-9, 31341288 PMC7015180

[ref3] Cao B. Liu Y. L. Wang N. Huang Y. Lu C. X. Li Q. Y. . (2025). Alterations of serum metabolic profile in major depressive disorder: a case-control study in the Chinese population. World J. Psychiatry 15:102618. doi: 10.5498/wjp.v15.i5.102618, 40495854 PMC12146990

[ref4] Casarotto P. C. Girych M. Fred S. M. Kovaleva V. Moliner R. Enkavi G. . (2021). Antidepressant drugs act by directly binding to TRKB neurotrophin receptors. Cell 184, 1299–1313.e19. doi: 10.1016/j.cell.2021.01.034, 33606976 PMC7938888

[ref5] Cavaleri D. Bassetti C. Cucchi G. De Fazio P. De Filippis R. Albert U. . (2026). Metabolomics biomarkers for precision psychiatry. Front. Psych. 17:1736206. doi: 10.3389/fpsyt.2026.1736206, 41767152 PMC12946017

[ref6] Chen Q. Jia T. Wu X. Chen X. Wang J. Ba Y. (2023). Polygalae radix oligosaccharide esters may relieve depressive-like behavior in rats with chronic unpredictable mild stress via modulation of gut microbiota. Int. J. Mol. Sci. 24:13877. doi: 10.3390/ijms241813877, 37762181 PMC10530649

[ref7] Cipriani A. Furukawa T. A. Salanti G. Chaimani A. Atkinson L. Z. Ogawa Y. . (2018). Comparative efficacy and acceptability of 21 antidepressant drugs for the acute treatment of adults with major depressive disorder: a systematic review and network meta-analysis. Lancet 391, 1357–1366. doi: 10.1016/S0140-6736(17)32802-7, 29477251 PMC5889788

[ref8] Dajas F. Abin-Carriquiry J. A. Arredondo F. Blasina F. Echeverry C. Martínez M. . (2015). Quercetin in brain diseases: potential and limits. Neurochem. Int. 89, 140–148. doi: 10.1016/j.neuint.2015.07.002, 26160469

[ref9] Ding W. Wang L. Li L. Li H. Wu J. Zhang J. . (2024). Pathogenesis of depression and the potential for traditional Chinese medicine treatment. Front. Pharmacol. 15:1407869. doi: 10.3389/fphar.2024.1407869, 38983910 PMC11231087

[ref10] Du Y. Ruan J. Zhang L. Fu F. (2020). Jieyu Anshen granule, a Chinese herbal formulation, exerts effects on poststroke depression in rats. Evid. Based Complement. Alternat. Med. 2020:7469068. doi: 10.1155/2020/7469068, 32184899 PMC7060433

[ref11] Duan J. Huang Y. Tan X. Chai T. Wu J. Zhang H. . (2021). Characterization of gut microbiome in mice model of depression with divergent response to escitalopram treatment. Transl. Psychiatry 11:303. doi: 10.1038/s41398-021-01428-1, 34016954 PMC8138009

[ref12] Dziedzic A. Maciak K. Bliźniewska-Kowalska K. Gałecka M. Kobierecka W. Saluk J. (2024). The power of psychobiotics in depression: a modern approach through the microbiota-gut-brain axis: a literature review. Nutrients 16:1054. doi: 10.3390/nu16071054, 38613087 PMC11013390

[ref13] Gao M. Wang J. Liu P. Tu H. Zhang R. Zhang Y. . (2023). Gut microbiota composition in depressive disorder: a systematic review, meta-analysis, and meta-regression. Transl. Psychiatry 13:379. doi: 10.1038/s41398-023-02670-5, 38065935 PMC10709466

[ref14] Hamilton P. J. Chen E. Y. Tolstikov V. Peña C. J. Picone J. A. Shah P. . (2020). Chronic stress and antidepressant treatment alter purine metabolism and beta oxidation within mouse brain and serum. Sci. Rep. 10:18134. doi: 10.1038/s41598-020-75114-5, 33093530 PMC7582177

[ref15] Hao W. Ma Q. Wang L. Yuan N. Gan H. He L. . (2024). Gut dysbiosis induces the development of depression-like behavior through abnormal synapse pruning in microglia-mediated by complement C3. Microbiome 12:34. doi: 10.1186/s40168-024-01756-6, 38378622 PMC10877840

[ref16] Jia X. Chen J. Huang R. Wang D. Wang X. (2023). Effect-enhancing and toxicity-reducing effects of Chaihu Jia Longgu Muli decoction in the treatment of multimorbidity with depression: a systematic review and meta-analysis. Pharm. Biol. 61, 1094–1106. doi: 10.1080/13880209.2023.2228356, 37439185 PMC10348031

[ref17] Li B. Xu M. Wang Y. Feng L. Xing H. Zhang K. (2023). Gut microbiota: a new target for traditional Chinese medicine in the treatment of depression. J. Ethnopharmacol. 303:116038. doi: 10.1016/j.jep.2022.116038, 36529248

[ref18] Liu X. Luo M. Wang Z. Yang S. J. Su M. Wang Y. . (2024). Mind shift I: Fructus Aurantii—Rhizoma chuanxiong synergistically anchors stress-induced depression-like behaviours and gastrointestinal dysmotility cluster by regulating psycho-immune-neuroendocrine network. Phytomedicine 128:155324. doi: 10.1016/j.phymed.2023.155324, 38552437

[ref19] Liu L. Wang H. Chen X. Zhang Y. Zhang H. Xie P. (2023). Gut microbiota and its metabolites in depression: from pathogenesis to treatment. EBioMedicine 90:104527. doi: 10.1016/j.ebiom.2023.104527, 36963238 PMC10051028

[ref20] Liu T. Xue Y. Wang L. Zhao N. Zhao T. Xie K. . (2026). Plasma profiles of carnitine and acylcarnitines in first-diagnosed, drug-naïve patients with depression: a case-control analysis. Behav. Brain Res. 497:115864. doi: 10.1016/j.bbr.2025.115864, 41086960

[ref21] Lv M. Wang Y. Qu P. Li S. Yu Z. Qin X. . (2021). A combination of cecum microbiome and metabolome in CUMS depressed rats reveals the antidepressant mechanism of traditional Chinese medicines: a case study of Xiaoyaosan. J. Ethnopharmacol. 276:114167. doi: 10.1016/j.jep.2021.114167, 33984458

[ref22] Ma R. H. Yang J. Qi L. W. Xin G. Z. Wang C. Z. Yuan C. S. . (2012). In vivo microdialysis with LC-MS for analysis of spinosin and its interaction with cyclosporin a in rat brain, blood and bile. J. Pharm. Biomed. Anal. 61, 22–29. doi: 10.1016/j.jpba.2011.11.014, 22169469

[ref23] Mahmoudiandehkordi S. Bhattacharyya S. Brydges C. R. Jia W. Fiehn O. Rush A. J. . (2022). Gut microbiome-linked metabolites in the pathobiology of major depression with or without anxiety-a role for bile acids. Front. Neurosci. 16:937906. doi: 10.3389/fnins.2022.937906, 35937867 PMC9350527

[ref24] Markov D. D. Novosadova E. V. (2022). Chronic unpredictable mild stress model of depression: possible sources of poor reproducibility and latent variables. Biology (Basel) 11:1621. doi: 10.3390/biology11111621, 36358321 PMC9687170

[ref25] Qiao L. Liu Y. Chen X. Xie J. Zhang Y. Yang K. . (2016). A HPLC-MS/MS method for determination of 6'''-feruloylspinosin in rat plasma and tissues: pharmacokinetics and tissue distribution study. J. Pharm. Biomed. Anal. 121, 77–83. doi: 10.1016/j.jpba.2016.01.005, 26780157

[ref26] Rong J. Wang X. Cheng P. Li D. Zhao D. (2025). Global, regional and national burden of depressive disorders and attributable risk factors, from 1990 to 2021: results from the 2021 global burden of disease study. Br. J. Psychiatry 227, 688–697. doi: 10.1192/bjp.2024.266, 39809717

[ref27] Ru J. Li P. Wang J. Zhou W. Li B. Huang C. . (2014). TCMSP: a database of systems pharmacology for drug discovery from herbal medicines. J. Cheminform. 6:13. doi: 10.1186/1758-2946-6-13, 24735618 PMC4001360

[ref28] Wang Y. Chen H. Feng P. Wang D. Du X. (2025). Traditional uses, nutritional properties, phytochemical metabolites, pharmacological properties, and potential applications of *Lilium* spp.: a systematic review. Front. Pharmacol. 16:1713957. doi: 10.3389/fphar.2025.1713957, 41368573 PMC12682905

[ref29] Wang X. Chen J. Zhang H. Huang Z. Zou Z. Chen Y. . (2019). Immediate and persistent antidepressant-like effects of Chaihu-jia-Longgu-Muli-tang are associated with instantly up-regulated BDNF in the hippocampus of mice. Biosci. Rep. 39:BSR20181539. doi: 10.1042/BSR20181539, 30473537 PMC6328878

[ref30] Wang Z. Wang X. Mou X. Wang C. Sun Y. Wang J. (2024). *Rehmannia glutinosa DC.-Lilium lancifolium Thunb.* in the treatment of depression: a comprehensive review and perspectives. Front. Pharmacol. 15:1471307. doi: 10.3389/fphar.2024.1471307, 39539631 PMC11557470

[ref31] Yan Y. Zhao N. Liu J. Zhang S. Zhang Y. Qin X. . (2025). Ziziphi Spinosae semen flavonoid ameliorates hypothalamic metabolism and modulates gut microbiota in chronic restraint stress-induced anxiety-like behavior in mice. Foods 14:828. doi: 10.3390/foods14050828, 40077533 PMC11898499

[ref32] Yang J. Zheng P. Li Y. Wu J. Tan X. Zhou J. . (2020). Landscapes of bacterial and metabolic signatures and their interaction in major depressive disorders. Sci. Adv. 6:eaba8555. doi: 10.1126/sciadv.aba8555, 33268363 PMC7710361

[ref33] Zhang G. Hu Q. Zou H. (2026). Bridging reward and resilience: the endocannabinoid system as a unifying mechanism in exercise-induced protection against major depressive disorder. Front. Psych. 17:1766980. doi: 10.3389/fpsyt.2026.1766980, 41877874 PMC13006632

[ref34] Zhao M. Che Y. Gao Y. Zhang X. (2024). Application of multi-omics in the study of traditional Chinese medicine. Front. Pharmacol. 15:1431862. doi: 10.3389/fphar.2024.1431862, 39309011 PMC11412821

[ref35] Zhao H. Jin K. Jiang C. Pan F. Wu J. Luan H. . (2022). A pilot exploration of multi-omics research of gut microbiome in major depressive disorders. Transl. Psychiatry 12:8. doi: 10.1038/s41398-021-01769-x, 35013099 PMC8748871

[ref36] Zhao Y. Xu D. Wang J. Zhou D. Liu A. Sun Y. . (2023). The pharmacological mechanism of chaihu-jia-longgu-muli-tang for treating depression: integrated meta-analysis and network pharmacology analysis. Front. Pharmacol. 14:1257617. doi: 10.3389/fphar.2023.1257617, 37808199 PMC10551636

[ref37] Zhao J. Zhang X. Dong L. Wen Y. Zheng X. Zhang C. . (2015). Cinnamaldehyde inhibits inflammation and brain damage in a mouse model of permanent cerebral ischaemia. Br. J. Pharmacol. 172, 5009–5023. doi: 10.1111/bph.13270, 26234631 PMC4621990

[ref38] Zhou X. Li Y. Yang Y. Wei L. Wang C. Xu J. . (2025). Regulatory effects of *Poria cocos* polysaccharides on gut microbiota and metabolites: evaluation of prebiotic potential. NPJ Sci. Food 9:53. doi: 10.1038/s41538-025-00416-9, 40263347 PMC12015419

